# Persistently elevated T cell interferon-γ responses after treatment for latent tuberculosis infection among health care workers in India: a preliminary report

**DOI:** 10.1186/1745-6673-1-7

**Published:** 2006-05-23

**Authors:** Madhukar Pai, Rajnish Joshi, Sandeep Dogra, Deepak K Mendiratta, Pratibha Narang, Keertan Dheda, Shriprakash Kalantri

**Affiliations:** 1 Division of Epidemiology, School of Public Health, University of California, Berkeley, USA; 2 Departments of Medicine & Microbiology, Mahatma Gandhi Institute of Medical Sciences, Sevagram, India; 3 Division of Pulmonary & Critical Care Medicine, San Francisco General Hospital, University of California, San Francisco, USA; 4 Centre for Infectious Diseases and International Health The Royal Free and University College Medical School, London, UK

## Abstract

**Background:**

T cell-based interferon-γ (IFN-γ) release assays (IGRAs) are novel tests for latent tuberculosis infection (LTBI). It has been suggested that T cell responses may be correlated with bacterial burden and, therefore, IGRAs may have a role in monitoring treatment response. We investigated IFN-γ responses to specific TB antigens among Indian health care workers (HCWs) before, and after LTBI preventive therapy.

**Methods:**

In 2004, we established a cohort of HCWs who underwent tuberculin skin testing (TST) and a whole-blood IGRA (QuantiFERON-TB-Gold *In-Tube *[QFT-G], Cellestis Ltd, Victoria, Australia) at a rural hospital in India. HCWs positive by either test were offered 6 months of isoniazid (INH) preventive therapy. Among the HCWs who underwent therapy, we prospectively followed-up 10 nursing students who were positive by both tests at baseline. The QFT-G assay was repeated 4 and 10 months after INH treatment completion (i.e. approximately 12 months and 18 months after the initial testing). IFN-γ responses to ESAT-6, CFP-10 and TB7.7 peptides were measured using ELISA, and IFN-γ ≥0.35 IU/mL was used to define a positive QFT-G test result.

**Results:**

All participants (N = 10) reported direct contact with smear-positive TB patients at baseline, during and after LTBI treatment. All participants except one started treatment with high baseline IFN-γ responses (median 10.0 IU/mL). The second QFT-G was positive in 9 of 10 participants, but IFN-γ responses had declined (median 5.0 IU/mL); however, this difference was not significant (*P *= 0.10). The third QFT-G assay continued to be positive in 9 of 10 participants, with persistently elevated IFN-γ responses (median 7.9 IU/mL; *P *= 0.32 for difference against baseline average).

**Conclusion:**

In an environment with ongoing, intensive nosocomial exposure, HCWs had strong IFN-γ responses at baseline, and continued to have persistently elevated responses, despite LTBI treatment. It is plausible that persistence of infection and/or re-infection might account for this phenomenon. Our preliminary findings need confirmation in larger studies in high transmission settings. Specifically, research is needed to study T cell kinetics during LTBI treatment, and determine the effect of recurrent exposures on host cellular immune responses.

## Background

The World Health Organization (WHO) has estimated that approximately one third of the world's population is infected with *Mycobacterium tuberculosis *[1,2]. This large pool of individuals with latent infection poses a major hurdle for global tuberculosis (TB) control efforts. Between 8 and 9 million people develop TB disease each year, and about 2 million die from TB every year [1,3].

Until recently, the only tool available to detect latent tuberculosis infection (LTBI) was the tuberculin skin test (TST). Although the TST is useful in clinical practice, it has several known limitations, including variable specificity, cross-reactivity with BCG vaccine and non-tuberculous mycobacterial (NTM) infection, and problems with reliability [4-6].

Because of advances in molecular biology and genomics, for the first time, an alternative has emerged in the form of a new class of T cell-based, *in vitro *assays that measure interferon-γ (IFN-γ) released by sensitized T cells after stimulation by *Mycobacterium tuberculosis *antigens [7-10]. Early versions of IFN-γ release assays (IGRAs) used purified protein derivative (PPD) as the stimulating antigen, but these tests have been replaced by newer versions that use antigens (early secreted antigenic target 6 [ESAT-6] and culture filtrate protein 10 [CFP-10]) that are more specific to *M. tuberculosis *than PPD. These antigens, encoded by genes located within the region of difference 1 (RD1) segment of the *M. tuberculosis *genome, are more specific because they are not shared with any of the BCG vaccine strains or certain species of NTM [11,12].

Two IGRAs are now available as commercial kits: the T-SPOT.*TB*^® ^test (Oxford Immunotec, Oxford, UK), and the QuantiFERON^®^-TB Gold^® ^(Cellestis Ltd, Carnegie, Australia) assay. The QuantiFERON^®^-TB Gold (QFT-G) assay is available in two formats, a 24-well culture plate format (approved by the US Food and Drug Administration [FDA] [13]), and a newer, simplified *In Tube *format (not FDA approved as yet; but available in other countries [14]). The T-SPOT.*TB *test is currently CE marked for use in Europe.

Research evidence, extensively reviewed elsewhere [7-10,13-15], suggests that IGRAs are more specific than TST, better correlated with markers of TB exposure in low incidence settings, and less affected by BCG vaccination than the TST. In the US, the Centers for Disease Control and Prevention (CDC) has recommended that QFT-G can be used in place of the TST for all indications, including screening of contacts, immigrants, and health care workers [13]. In the UK, the National Institute for Health and Clinical Excellence (NICE) Tuberculosis guidelines recommend a two-step strategy for LTBI diagnosis: initial screen with TST, and those who are positive (or in whom TST may be unreliable) should then be considered for IGRA testing, if available, to confirm positive TST results [16].

Although IGRAs are promising and offer several logistical advantages [7,8,13,14], unresolved issues remain [7,8,10,13,17,18]. One area of controversy is whether they can be used for monitoring treatment responses [7,8,10]. It has been hypothesized that short incubation assays (e.g. both commercial assays use 16 – 24 hours incubation) detect responses of partially activated, effector T cells that have recently encountered antigens *in vivo*, and can therefore rapidly release IFN-γ when stimulated *in vitro *[19,20]. In contrast, long-lived central memory T cells that may persist even after clearance of the organism (e.g. previously treated TB) are less likely to release IFN-γ with short incubation [20]. Effector response may be driven by the antigen load, and there is evidence that reduction of the antigen load by treatment decreases T cell responses [19,20].

Although studies have examined the effect of active TB treatment on IFN-γ responses, the results have been inconsistent. As reviewed elsewhere [7,8], some studies have shown declining responses after treatment, whereas others have shown unchanging, fluctuating, or increasing responses over treatment. It is plausible that variations in incubation periods (short vs. long), antigens (proteins vs. peptides), and assay formats (ELISA vs. ELISPOT) might explain some of the discrepancies [7,8]. In contrast to active TB, limited data exist on T cell responses after LTBI preventive therapy [21,22]. In this preliminary report, we present data on T cell responses before and after preventive therapy among health care workers (HCWs) in rural India. TB is an important but poorly studied occupational risk among Indian HCWs [23-25]. As in the case of most developing countries, infection control measures are rarely used in Indian hospitals, and HCWs tend to get repeatedly exposed to smear-positive TB patients. However, not much research has been conducted using newer IGRAs among HCWs who work in high incidence countries.

## Methods

### Description of the study cohort

In early 2004, we established a cohort of 726 HCWs (median age, 22 years; 62% women) who underwent TST and QFT-G *In-Tube *testing at a rural medical school hospital in India [23]. This hospital has a high TB case load, and like most hospitals in India, repeated exposure to TB is common among HCWs [23]. Our cohort of 726 HCWs was comprised of 353 (49%) medical students and nursing students, 73 (10%) interns and residents, 161 (22%) nurses, 13 (2%) attending physicians/faculty, and 126 (17%) orderlies and laboratory workers. About 71% of the cohort had BCG vaccine scars, and only 5% had received TST prior to the baseline study. At baseline, of the 726 HCWs, 68% reported having had at least one direct contact with a patient with TB (direct contact was defined as contact between two people that is of sufficient distance to allow conversation between them [26]).

### Tuberculin skin test and QuantiFERON-TB Gold In Tube assay

At baseline, all HCWs underwent a TST (Mantoux technique) using 1 TU PPD RT23, the standard dose in India [27]. 1 TU of the PPD was administered on the volar surface of the forearm by a certified technician using the Mantoux method. The maximum transverse diameter of the induration was read after 48 – 72 hours using a blinded caliper. IFN-γ responses to ESAT-6, CFP-10, and TB7.7 (Rv2654) were measured by the QFT-G In Tube assay, as per the manufacturer's instructions (Cellestis Limited, Victoria, Australia).

The QFT-G In Tube assay involved two stages: (1) incubation of whole blood with antigens, and (2) measurement of IFN-γ production in harvested plasma by ELISA. Venous blood was directly collected into three 1 mL heparin-containing tubes. One tube contained only heparin as negative control, another also contained the T-cell mitogen phytohemagglutinin as positive control, and the third tube had overlapping peptides representing the entire sequences of ESAT-6 and CFP-10 and another peptide from the TB antigen TB7.7 (Rv2654). Within 2 – 6 hours of blood draw, the tubes were incubated at 37°C. After 24 hours of incubation, the tubes were centrifuged and plasma was harvested and frozen at -70°C until the ELISA was performed. The amount of IFN-γ was quantified using the QFT ELISA. The ELISA readout was analyzed using the QFT-G software. IFN-γ values (International Units [IU] per mL) for TB-specific antigens and mitogen were corrected for background by subtracting the value obtained for the respective negative control.

As recommended by the manufacturer and used in previous studies [23,24,28-30], an IFN-γ ≥0.35 IU/mL for (TB antigens – Negative control) was considered indicative of TB infection. For a QFT-G result to be valid, the (Mitogen – Negative control) must be = 0.5 IU/mL and/or (TB antigens – Negative control) must be ≥ 0.35 IU/mL. All assays were deemed valid, and met the internal quality standards. No indeterminate results were reported.

### Cohort follow-up

As reported previously [23], at baseline (in 2004), valid TST results were available for 720 of 726 HCWs, and valid QFT-G results were available for 725 of 726 HCWs. The baseline prevalence estimates of TST and QFT-positivity were comparable (41% [95% CI 38% – 45%] and 40% [95% CI 37% – 43%], respectively). Baseline agreement between TST and QFT-G was high (81%, κ = 0.61 [95% CI 0.56–0.67]). Increasing age and years in the health profession were significant risk factors for both QFT and TST positivity. Since 2004, this HCW cohort has been under follow-up. Notably, a repeat survey of this cohort in 2005 showed a high annual risk of TB infection (ARTI) among young trainees at this hospital [24]; the ARTI was estimated to be 5%, and this rate is higher than the population average of 1.5% for India [31].

After baseline testing in 2004, individuals positive by either TST or QFT-G were offered 6 months of standard isoniazid (INH) as preventive therapy. At baseline, 360 of 726 (50%) HCWs were positive by either TST or QFT-G [23]. However, only 61 (17%) accepted INH, and 35 (10%) completed treatment.

In January 2005, 22 nursing students who had undergone baseline testing in January/February 2004, and had completed INH treatment 4 months earlier, underwent a follow-up QFT-G. These students underwent a third QFT-G in July 2005, 10 months after treatment. Of the 22 students, 10 students were TST and QFT-G positive at baseline (i.e. TST+/QFT-G+), and underwent both follow-up QFT-G tests. These students also completed questionnaires on their work patterns and TB exposure during the follow-up. Identical assay protocols were used for all QFT-G tests, and follow-up assays were performed blinded to the previous results. Because these HCWs were TST-positive at baseline, they were not asked to undergo repeat tuberculin skin testing. All participants gave informed consent, and the research protocol was approved by ethics committees in India and USA [23].

### Statistical analyses

Data were analyzed using Stata 9 (Stata Corp, Texas, USA). The main outcome was effect of treatment on QFT-G results, expressed as dichotomous (positive/negative) and continuous measures (IFN-γ expressed as IU/mL). Because the QFT-G ELISA cannot accurately measure IFN-γ values >10 IU/mL, values >10 IU/mL were treated as 10 IU/mL in all the analyses. Differences between average IFN-γ levels were analyzed using the Wilcoxon signed-rank test.

## Results

All the participants (N = 10) were female nursing students (median age 19 years, range 18 to 24), and reported direct contact (pragmatically defined as contact between two people that is of sufficient distance to allow conversation between them [23,26]) with smear-positive TB patients at baseline, during and after LTBI treatment. All participants were TST and QFT-G positive at baseline (in 2004), and all had completed INH treatment with good adherence. The median baseline TST induration was 17 mm (range 14 to 20 mm), and 6 of 10 (60%) participants had BCG scars.

As seen in the Table and Figure, all participants except one started treatment with high baseline IFN-γ responses (median 10.0 IU/mL, after correcting for the background). The second QFT-G assay was positive in 9 of 10 (90%) participants, but IFN-γ responses had declined (median 5.0 IU/mL); however, this difference was not statistically significant (*P *= 0.10). The third QFT-G assay continued to be positive in 9 of 10 (90%) participants, with persistently elevated IFN-γ responses (median 7.9); *P *= 0.32 for difference against the baseline average. Only one participant had a reversion; this individual had a baseline response of 0.39 IU/mL, just above the diagnostic threshold of 0.35 IU/mL.

## Discussion

In latent TB infection, bacterial burden is low, and preventive therapy with a single drug (isoniazid) for 6–9 months is considered adequate to prevent active disease [32,33]. However, currently there is no test or surrogate marker to monitor response to LTBI preventive therapy. Also, there is no reliable test that can detect new exogenous TB infection after successful clearance of the original infection. An association between INH treatment and tuberculin reversions has been reported in the past [34,35], and based on the results of some studies that showed declining IFN-γ responses after active TB treatment [36-39], it is plausible that INH treatment might decrease IFN-γ responses. In contrast to active TB, the kinetics of T cell responses during LTBI treatment has not been well studied, and, to our knowledge, this is the first report of longitudinal changes in IFN-γ responses after LTBI treatment among HCWs in a high burden, developing country, measured using the latest *In Tube *version of the QFT-G assay.

In a recent study from Japan, 37 HCWs underwent QFT-G assays after INH for 6 months [21]. Although many HCWs continued to remain QFT-G positive, IFN-γ levels were significantly lower soon after treatment: ESAT-6 responses decreased from 3.36 ± 4.97 to 1.21 ± 2.23 IU/mL; CFP-10 responses decreased from 1.69 ± 3.15 to 0.23 ± 2.04 IU/mL [21]. The authors speculated that it might take longer than 6 months for clearance of infection, and recommended longer follow-up.

In another recent study, healthy TST-positive immigrants in the UK underwent an ELISPOT assay at the beginning, during, and end of INH and rifampicin for 12 weeks [22]. Treatment resulted in a 1.8 fold rise in the numbers of IFN-γ producing T cells within 26 ± 4 days of starting treatment, followed by a decrease to below baseline by the end of treatment [22]. There was no significant overall change in T cell responses in an untreated group.

Our study, although limited by the small numbers, and lack of an untreated control group, offers a different perspective, because it was conducted in a nosocomial setting in a high-prevalence country. Almost all HCWs in our study had persistent high T cell IFN-γ responses, even 10 months after treatment completion. Although there was a modest decline in the average IFN-γ responses after treatment, the post-treatment responses remained substantially higher than the diagnostic threshold. It is possible that some of the observed changes were due to regression to the mean, or to random variability. The only participant who had a reversion, had a low baseline IFN-γ response, close to the threshold. This finding is consistent with our previous follow-up study of this HCW cohort [24]. In the absence of any longitudinal data on outcomes, it is not clear if individuals with high T cell responses after treatment are at greater or lesser risk of progressing to disease. Long-term follow-up of large cohorts are needed to answer these questions.

In the absence of a strong evidence base on this topic, we put forth several tentative hypotheses that might explain our findings. First, in high incidence settings, individuals with LTBI may have very strong IFN-γ responses at diagnosis. In fact, 80% of our participants had baseline values ≥10 IU/mL, much higher than those reported from Japan [21]. Therefore, unless the IFN-γ levels drop drastically, QFT-G reversions are unlikely, even after treatment. Second, it is possible, that in HCWs repeatedly exposed to TB, 6 months of INH is inadequate to clear the infection. INH resistance might influence this. In India, about 10% of newly diagnosed smear-positive TB patients have INH resistance [40]. It will be interesting to do similar experiments after the longer, 9 month INH regimen, or alternative LTBI regimens (e.g. rifampin for 4 months).

Third, ongoing exposure and/or exogenous re-infection might keep the effector T cells partially activated, and therefore T cell responses remain robust even after antigen load declines with therapy. Interestingly, some individuals had a transient decline in T cell responses shortly after treatment, but much higher responses subsequently. It is plausible that these individuals became re-infected after successful clearance of their primary infection. Studies in high incidence settings are required to address these intriguing questions, but will be challenging to conduct because of potential confounders such as malnutrition, BCG vaccination, non-tuberculous mycobacteria, and tropical infections (e.g. helminthiasis). In low incidence settings (e.g. Japan and UK), it is possible that treatment might cause steeper declines in IFN-γ responses [21,22]. Fourth, the duration of follow-up in our study may have been inadequate. It might take longer for IFN-γ levels to decline to negativity after treatment [21]. However, in high incidence countries where recurrent exposure is widespread, IFN-γ responses may not disappear, even with longer follow-up. Fifth, exposure to certain non-tuberculous (environmental) mycobacteria (e.g. *M. marinum*) may play a role in inducing T cell responses, even after clearance of the original *M. tuberculosis *infection [39]. All of these hypotheses deserve further study, particularly in high incidence settings.

In conclusion, our preliminary study, although limited by numbers, raises the interesting hypothesis that in an environment with ongoing, intensive nosocomial exposure, HCWs may have high IFN-γ responses at baseline, and continue to have persistently elevated T cell responses, despite LTBI treatment. It is possible, although unproven as yet, that persistence of infection and/or re-infection might account for this phenomenon, and raises concerns about efficacy of conventional preventive therapy in high incidence settings with recurrent exposure. Further research is needed to study T cell kinetics during LTBI treatment, and determine the effect of repeated exposures on host cellular immune responses, particularly in high incidence settings. The availability of standardized, commercial T cell-based IGRAs might greatly facilitate such novel investigations.

## Abbreviations

BCG: bacille Calmette-Guerin

CFP10: culture filtrate protein 10

ESAT-6: early secreted antigenic target 6

HCW: health care worker

IGRA: Interferon-γ release assay

IFN-γ: interferon-γ

INH: isoniazid

LTBI: latent tuberculosis infection

NTM: non-tuberculous mycobacteria

PPD: purified protein derivative

QFT-G: QuantiFERON-TB Gold

RD1: region of difference 1

TB: tuberculosis

TST: tuberculin skin test

## Competing interests

The author(s) declare that they have no competing interests.

## Authors' contributions

MP conceived and designed the study, raised funding support, participated in its supervision, performed the data analyses and drafted the manuscript. RJ carried out the field work and interviews, participated in the analysis and helped revise the manuscript. SD assisted in carrying out the immunoassays, and revised the manuscript. DK and PN participated in the study design, provided technical support, and supervision of the laboratory work. KD provided technical assistance with interpretation of immunology data, and helped draft the manuscript. SK conceived of the study, and participated in its design and supervision, and helped to draft the manuscript. All authors read and approved the final manuscript.

**Figure 1:**
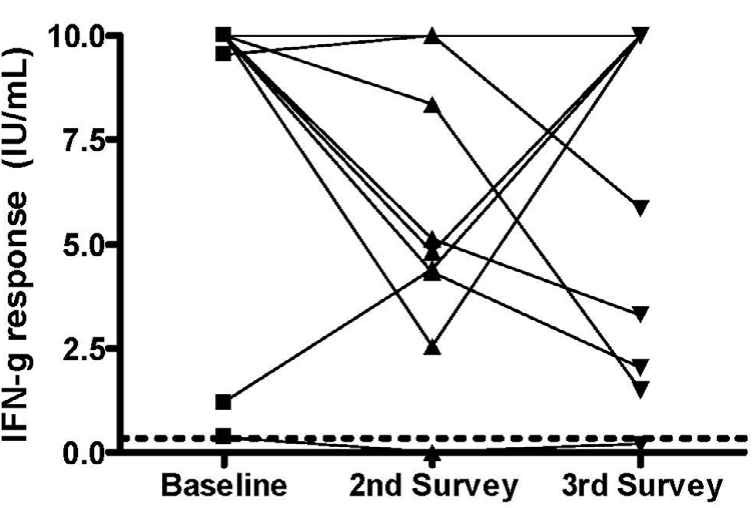
**Pre-treatment and post-treatment interferon-γ responses in nursing students treated with isoniazid preventive therapy for 6 months (N = 10)**. Baseline interferon-γ (IFN-γ) levels were measured using the QuantiFERON-TB Gold In Tube assay at the time of latent tuberculosis infection diagnosis (January/February 2004). The second measurement was made in January 2005, 4 months after isoniazid (INH) preventive therapy completion. The third measurement was made in July 2005, 10 months after INH treatment completion. All subjects were positive by the QuantiFERON-TB Gold In Tube assay and tuberculin skin test at baseline. The QuantiFERON-TB Gold diagnostic cut-point of 0.35 IU/mL is shown as a horizontal dotted line. IFN-γ levels >10.0 IU/mL have been shown as 10 IU/mL

**Table 1:** T cell interferon-γ responses in nursing students treated for latent tuberculosis infection with isoniazid preventive therapy for 6 months (N = 10)

ID number	Age at baseline	Sex M/F	BCG scar (Y/N)	TST at baseline (mm)	IFN-γ responses at baseline (Jan/Feb 2004)		IFN-γ responses at second survey, 4 months after INH treatment (Jan 2005)		IFN-γ responses at third survey, 10 months after INH treatment (July 2005)	
					
					Result (Pos/Neg)*	IU/mL**	Result (Pos/Neg)*	IU/mL**	Result (Pos/Neg)*	IU/mL**
10055	19	F	Y	14	P	10.0	P	10.0	P	10.0
10066	19	F	Y	15	P	10.0	P	2.55	P	10.0
10069	19	F	Y	17	P	9.55	P	10.0	P	10.0
10077	19	F	N	19	P	10.0	P	4.34	P	2.04
10082	19	F	Y	18	P	10.0	P	10.0	P	5.86
10089	19	F	Y	16	P	10.0	P	8.35	P	1.51
10093	19	F	N	20	P	10.0	P	5.13	P	3.31
10115	24	F	Y	17	P	0.39	N	0	N	0.21
10282	19	F	N	15	P	1.21	P	4.40	P	10.0
10290	18	F	N	18	P	10.0	P	4.81	P	10.0
**Mean (SD)**						**8.1 (3.9)**		**5.9 (3.5)**		**6.3 (4.2)**
**Median**						**10.0**		**5.0**		**7.9**

*QFT-G cut-point for positivity: ≥0.35 IU/mL

** IFN-γ values >10.0 IU/mL have been shown as 10 IU/mL

TST: tuberculin skin test; QFT-G: QuantiFERON-TB Gold In Tube assay; INH: isoniazid; BCG: bacille Calmette-Guerin; SD: standard deviation
